# Reproducibility and robustness of motor cortical stimulation to assess muscle relaxation kinetics

**DOI:** 10.14814/phy2.15491

**Published:** 2022-10-20

**Authors:** Joery P. Molenaar, Elianne van Zandvoort, Baziel G. van Engelen, Nicol C. Voermans, Jonne Doorduin

**Affiliations:** ^1^ Department of Neurology, Donders Institute for Brain, Cognition and Behaviour Radboud University Medical Center Nijmegen The Netherlands; ^2^ Department of Neurology Rijnstate Arnhem The Netherlands

**Keywords:** muscle relaxation, reproducibility, robustness, skin temperature, transcranial magnetic stimulation

## Abstract

Transcranial magnetic stimulation (TMS) of the motor cortex can be used during a voluntary contraction to inhibit corticospinal drive to the muscle and consequently induce involuntary muscle relaxation. Our aim was to evaluate the reproducibility and the effect of varying experimental conditions (robustness) of TMS‐induced muscle relaxation. Relaxation of deep finger flexors was assessed in 10 healthy subjects (5 M, 5 F) using handgrip dynamometry with normalized peak relaxation rate as main outcome measure, that is, peak relaxation rate divided by (voluntary plus TMS‐evoked)force prior to relaxation. Both interday and interrater reliability of relaxation rate were high with intraclass correlation coefficient of 0.88 and 0.92 and coefficient of variation of 3.8 and 3.7%, respectively. Target forces of 37.5% of maximal voluntary force or higher resulted in similar relaxation rate. From 50% of maximal stimulator output and higher relaxation rate remained the same. Only the most lateral position (>2 cm from the vertex) rendered lower relaxation rate (mean ± SD: 11.1 ± 3.0 s^−1^, 95% CI: 9.0–13.3 s^−1^) compared to stimulation at the vertex (12.8 ± 1.89 s^−1^, 95% CI: 11.6–14.1 s^−1^). Within the range of baseline skin temperatures, an average change of 0.5 ± 0.2 s^−1^ in normalized peak relaxation rate was measured per 1°C change in skin temperature. In conclusion, interday and interrater reproducibility and reliability of TMS‐induced muscle relaxation of the finger flexors were high. Furthermore, this technique is robust with limited effect of target force, stimulation intensity, and coil position. Muscle relaxation is strongly affected by skin temperature; however, this effect is marginal within the normal skin temperature range. We deem this technique well suited for clinical and scientific assessment of muscle relaxation.

## Graphical abstract

Transcranial magnetic stimulation (TMS) can be used to assess muscle relaxation during a voluntary contraction by abruptly halting neural drive to the muscle. Our results show that this technique has high interday and interrater reproducibility and reliability. Furthermore, generated results are robust with limited effect of target force, stimulation intensity, coil position, and skin temperature on relaxation rate. TMS‐induced muscle relaxation appears well suited for clinical and scientific assessment of muscle relaxation rate.

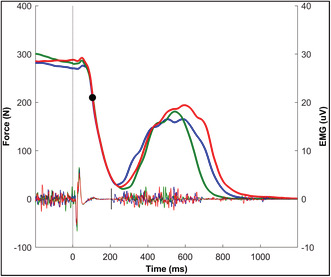

## 1 INTRODUCTION

Transcranial magnetic stimulation (TMS) is a non‐invasive technique that can be used to assess muscle relaxation kinetics (Todd et al., 2007). Stimulation of the primary motor cortex during a maximal voluntary muscle contraction (MVC) induces a transient cortical excitation causing a small increase in force (superimposed twitch), followed by cortical and spinal inhibition resulting in abrupt involuntary muscle relaxation (Barker et al., 1985; Fuhr et al., 1991; Inghilleri et al., 1993; Wilson et al., 1993). Electromyographically this corresponds to an initial motor‐evoked potential (MEP) followed by a silent period during which there is virtually no muscle activity (Barker et al., 1985; Day et al., 1989; Fuhr et al., 1991; Todd et al., 2005). The abrupt halt of neural input to the muscle allows for measurements of intrinsic muscle relaxation properties (i.e., without any voluntary/corticospinal influences).

TMS‐induced muscle relaxation can complement current physical and ancillary investigations by providing quantitative data on in vivo muscle relaxation kinetics which could lead to valuable insights in healthy subjects and in patients with impaired muscle relaxation, such as myotonic dystrophy, non‐dystrophic myotonias, and Brody disease (Kleine & Stegeman, 2007; Molenaar et al., 2018; Molenaar et al., 2020). For these myopathies, TMS‐induced muscle relaxation could be used as a diagnostic tool, monitor disease progression, and serve as an outcome measure in clinical trials. Furthermore, this technique can contribute in unraveling the underlying pathological mechanisms in myopathies with impaired muscle relaxation. We recently demonstrated this in nemaline myopathy type 6 by showing that in vivo muscle relaxation using TMS reflects in vitro relaxation kinetics of muscle fibers and myofibrils from biopsies (de Winter et al., 2020).

Muscle relaxation in healthy subjects has been studied previously using this technique, example, in elbow flexors (Molenaar et al., 2013; Todd et al., 2007), finger flexors (Molenaar et al., 2018), knee‐extensors (Vernillo et al., 2021), dorsiflexors, and plantar flexors (McNeil et al., 2013). Furthermore, physiological slowing effects of muscle cooling and fatigue on muscle relaxation have been demonstrated, as well as slower muscle relaxation in female compared to male and the elderly compared to younger adults (Hunter et al., 2008; McNeil et al., 2013; Molenaar et al., 2013; Todd et al., 2007).

Other methods of quantitative assessment of muscle relaxation include measurement of the relaxation phase of the resting twitch following electrical peripheral nerve or motor point stimulation (Mak et al., 1991; Senefeld et al., 2020). However, this does not measure muscle relaxation during voluntary contraction when muscle properties can be changing rapidly (Todd et al., 2007). With electrical stimulation it is difficult to test relaxation properties during maximal muscle contraction. This can only be achieved by the assessment of muscle relaxation following high frequency (tetanic) electrical stimulation which is very painful and remains only an approximation of a true MVC (De Ruiter, Wevers, et al., 1999). Alternatively, strong peripheral nerve stimulation during an MVC can halt nerve activity (silent period of peripheral nerve) resulting in involuntary abrupt muscle relaxation (McLellan, 1973). However, this electrically induced silent period is too short for the muscle to reach its peak relaxation rate. In other words, the muscle can contract again before maximum muscle relaxation rate is reached (Leis et al., 1991; McLellan, 1973). Muscle relaxation assessed by TMS can overcome these shortcomings by abruptly halting corticospinal drive to a voluntarily (sub)maximally activated muscle for up to 400 ms (Inghilleri et al., 1993).

Previously we demonstrated that TMS‐induced muscle relaxation results in very low variability in successive measurements of peak relaxation rate (i.e., high repeatability) and a small measurement error in relation to the differences between subjects (i.e., high reliability) (Molenaar et al., 2018). However, the variation in measurement by different raters (i.e., reproducibility) is currently unknown. TMS‐induced muscle relaxation of elbow flexors has previously been demonstrated to have high interday reproducibility (Todd et al., 2007). It is not known if this holds true for deep finger flexors. Furthermore, it is largely unknown if results are robust when measurements are performed under (slightly) varying experimental conditions (i.e., robustness). First, the magnitude of voluntary force that is reached prior to magnetic stimulation may affect muscle relaxation via selective recruitment of different types of motor units at different levels of activation (Kleine & Stegeman, 2007; Todd et al., 2007). Second, stimulation intensity is known to affect silent period duration and thus possibly peak relaxation rate (Inghilleri et al., 1993; Wilson et al., 1993). Third, a change in coil position on the scalp could potentially lead to suboptimal cortical inhibition thus underestimating peak relaxation rate. Fourth, muscle cooling and heating has been demonstrated to respectively decrease and increase muscle relaxation rate in human and animal muscle (Bennett, 1985; de Ruiter, Jones, et al., 1999; Hopf & Maurer, 1990; Molenaar et al., 2018; Ranatunga, 1982; Todd et al., 2005). However, the skin temperature range in which TMS‐induced muscle relaxation remains similar is unknown.

Accordingly, in the current study we aimed to show that TMS‐induced muscle relaxation rate in deep finger flexors is reproducible between days and raters and robust under varying experimental conditions, that is, the effects of target contractile force, stimulation intensity, TMS coil position, and skin temperature on relaxation rate.

## 2 MATERIALS AND METHODS

### 2.1 Subjects

This cross‐sectional physiological study was performed at the Radboud university medical center, Nijmegen, the Netherlands. Ten healthy subjects (5 male and 5 female) were included in this study. This sample size was deemed sufficient because of the previously demonstrated high repeatability and high reliability for TMS‐induced peak relaxation rate of the deep finger flexors (Molenaar et al., 2018). Furthermore, other similar physiological studies using TMS‐induced relaxation have demonstrated effects using similarly small sample sizes (McNeil et al., 2013; Todd et al., 2007).

Except for one male subject, all subjects were right‐handed, as determined by the Edinburgh Handedness Inventory (Oldfield, 1971). Exclusion criteria were the standard contra‐indications for TMS (Rossi et al., 2009), use of medication that possibly influences brain or muscle activity, and age above 65 years old, due to a possible slowing effect of age on muscle relaxation (Molenaar et al., 2013). Subjects were asked to refrain from strong physical exercise with hands and arms in the three days before a measurement session. Experiments were conducted after obtaining full understanding and written consent from the subjects. The study was approved by the local medical ethics committee (NL60169.091.16), and is conducted in accordance with the Declaration of Helsinki and its later amendments.

### 2.2 Questionnaire and physical examination

All participants answered a short questionnaire to determine handedness (Edinburgh Handedness Inventory). Physical activity was assessed by the General Practice Physical Activity Questionnaire: physical activity index of 1 = inactive, 2 = moderately inactive, 3 = moderately active, 4 = active (DoH, 2009; Oldfield, 1971). Height and body weight were measured and body mass index was calculated.

#### 2.2.1 Force and EMG recordings

**FIGURE 1:**
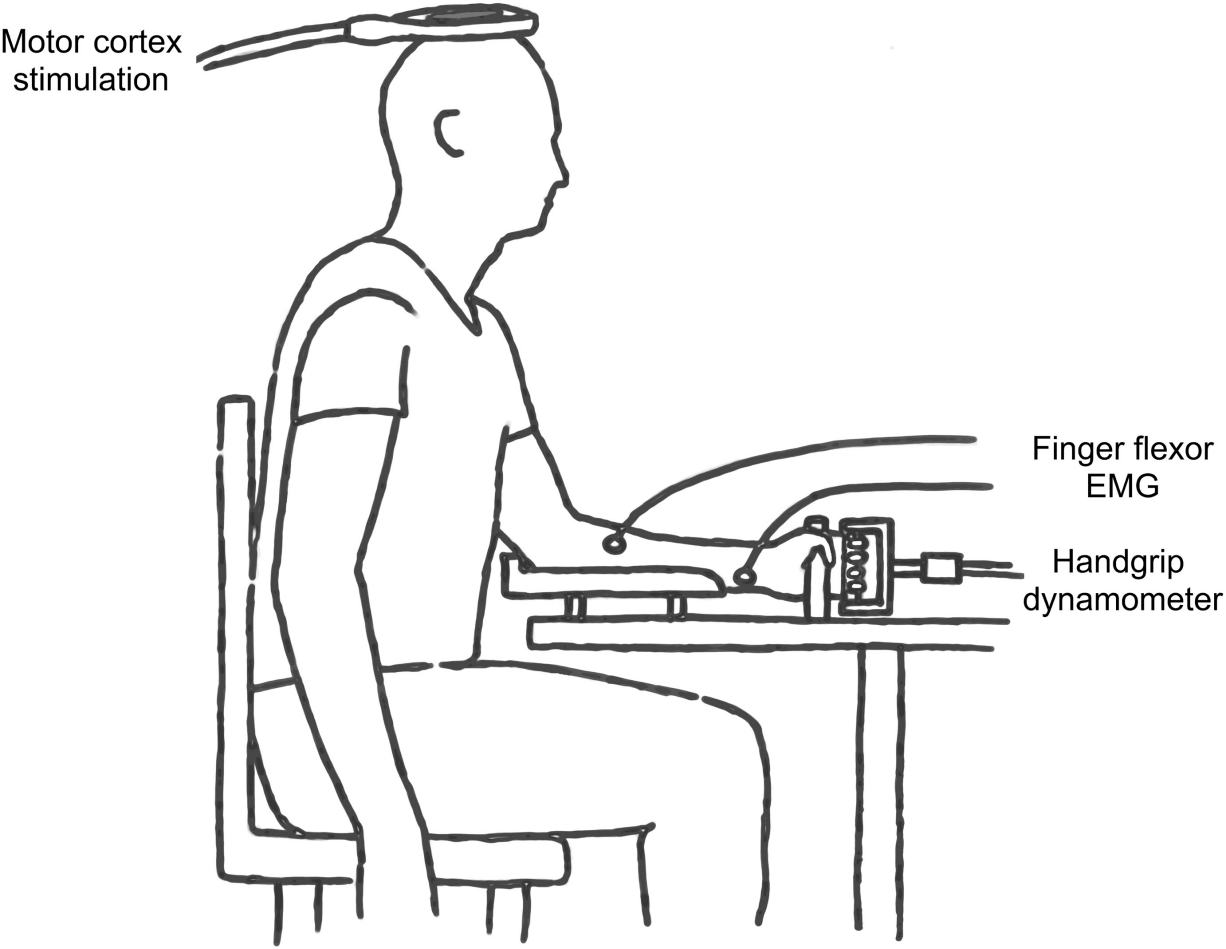
Line diagram of the experimental arrangement for testing of relaxation rate of deep finger flexors. Testing was performed on the dominant arm. This figure has previously been published (Molenaar et al., 2018). Abbreviation: EMG, electromyography.

Isometric finger flexor force was assessed using a handgrip dynamometer. The handgrip was set up such that only the distal phalanxes and part of the middle phalanxes could wrap around the distal part of the handgrip, thus mainly testing force of deep finger flexors. Subjects were seated comfortably with the elbow of their dominant arm in flexion and their forearm stabilized in a support (Figure 1). Force was measured by strain gauges in the handgrip device, recorded via an analog‐to‐digital converter (NI DAQPad‐6015, National Instruments) with a sampling frequency of 2 kHz, and low‐pass filtered at 50 Hz. Muscle activity of the finger flexor and extensor muscles was recorded using surface electromyography (EMG). Cloth hydrogel electrodes (H69P, Kendall Medical Supplies) were positioned with the recording electrode over the muscle belly and reference electrode over the distal tendon. Before placement of the electrodes, skin was cleansed with alcohol wipes and shaved if necessary. EMG signals were amplified with a 32‐channel amplifier (Porti System, Twente Medical Systems International BV), bandpass filtered (10–1024 Hz), and sampled with a frequency of 2048 Hz. Force and EMG signals were synchronized using TTL pulses. Exerted force was visualized real‐time and displayed on a screen placed directly in front of the participant (visual feedback). Force and EMG data were stored synchronously and visualized using custom in‐house developed software (Matlab, version 2014b, MathWorks).

Skin temperature was evaluated 1 cm distal to the finger flexors electrode using a handheld infrared thermometer (62 MAX, Fluke corporation).

### 2.3 Transcranial magnetic stimulation

TMS pulses were generated by a Magstim 200 (Magstim Company Ltd., Whitland, Wales). Pulses were applied with a circular coil (90 mm Ø) centred over the vertex unless specified otherwise. Current flow (anterior–posterior) was directed to stimulate the dominant hand (Groppa et al., 2012). With this orientation the cortical area that is mostly stimulated is actually ~5 cm lateral (over the contralateral hemisphere) from the centre of the circular coil (Barker et al., 1985). Participants wore a white bathing cap on which the position of the vertex was marked.

### 2.4 Experimental protocol

Data were collected in three recording sessions on three days. Each session lasted approximately 1.5 hours, including preparations.

Interday and interrater reproducibility and reliability were assessed. Furthermore, the protocol comprised multiple experiments, each addressing a separate experimental element: target contractile force (%MVC, maximal voluntary contraction), stimulus intensity (%MSO, maximal stimulator output, with 100% being the highest output setting of the magnetic stimulator, i.e., 2 Tesla), TMS coil position, and skin temperature (Table 1).

**TABLE 1:** Experimental protocol

Day	Experiment	Contractile force (%MVC)	Stim. Output (%MSO)	Coil position	Cooling/heating
1	Interday (Rater 1)	100	*	Vertex	No
	%MVC	12.5 ‐ 87.5	*	Vertex	No
	%MSO	100	40–100	Vertex	No
2	Interday + Interrater (Rater 1)	100	*	Vertex	No
	Interrater (Rater 2)	100	*	Vertex	No
	Coil position	50	*	Grid (19 positions)	No
3	Temperature	50	*	Vertex	Bath 10°C (15 min) Bath 40°C (10 min)

Abbreviations: MSO, maximal stimulator output; MVC, maximal voluntary contraction.

* %MSO needed to induce a silent period of ~200 ms.

In general, all measurements consisted of multiple brief (~2 s duration) maximal or sub‐maximal contractions of finger flexors. During the contractions, a TMS pulse was delivered by the rater when the targeted force level was reached (visually assessed). Participants were instructed to try to continue the contraction during and after the TMS pulse, in order to reliably estimate silent period duration (return of voluntary EMG after the TMS pulse).

*Preparation –* At the start of each session, subjects were asked to perform three brief maximal contractions, interleaved with one minute rest, in order to assess their maximal contraction strength for that recording session. Verbal encouragement was given during each MVC attempt. Next, the stimulator output needed to induce a silent period of approximately 200 ms was assessed. This was done at a target force of 30% of MVC and with increments of 10% of maximal stimulator output, until a silent period of 200 ms was achieved (estimated live using visual feedback of the EMG signal). This stimulator strength was then used during the session unless specified otherwise.

*Interday and interrater variation (day 1 and 2) –* on the first day, participants performed three consecutive MVCs with TMS‐induced muscle relaxation, interleaved with one minute rest. On the second day this block was repeated by the same rater to assess interday reliability. This was directly repeated by a different investigator on day 2, that is, three MVCs with TMS‐induced relaxation including the *preparation* process. For this latter part of the protocol (to study interrater reliability) only one investigator was present with the subject who was blinded from the other investigator regarding all procedures, including giving instructions, placement of EMG electrodes, measurement of the vertex, and operating the magnetic coil. The two raters alternated between subjects on performing the experiments of day 1. Force and EMG data were analyzed by one and the same rater as no variation was to be expected between raters when using the same Matlab algorithm.

*Target contractile force (day 1) –* The influence of target contractile force on muscle relaxation properties was assessed. Subjects were instructed to squeeze in the handgrip dynamometer at different percentages of their previously determined maximal force (12.5% – 87,5% of MVC in steps of 12.5%), aided by visual feedback (target line on the screen). When the target force was reached, a TMS pulse was administered on the vertex. Trials were interleaved with 50s rest. There were three contractions at every target force level in random order.

*Stimulation intensity (day 1) –* To assess the impact of stimulation intensity (%MSO) consecutive MVCs were performed (90s rest in between) with stimulator output varying between 40 and 100% MSO in steps of 10% (random order). There were two contractions at every stimulator output level in random order.

**FIGURE 2:**
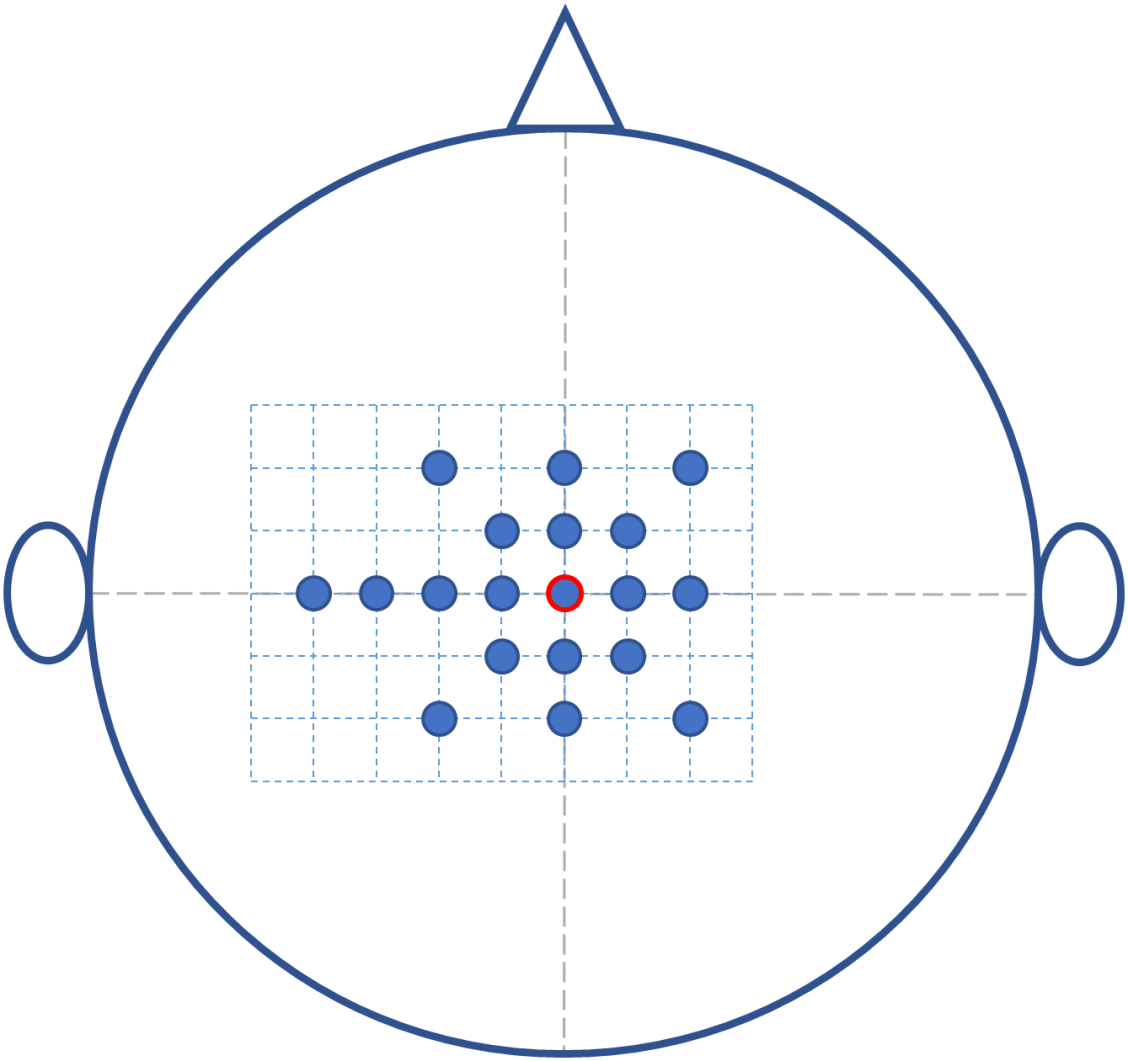
Schematic representation of the grid used for the coil position experiment, as seen from above for right‐handed subjects. The red circle represents the vertex. Each square (dashed lines) measures 1 × 1 cm

*Coil position (day 2) –* A grid was drawn on the bathing cap with 19 positions surrounding the vertex (Figure 2). In daily practice, it would be just as likely to accidentally defer 1‐2 cm to the left or the right of the vertex. Therefore, we aimed to test the effect of positioning the coil on either side of the vertex (also over the ipsilateral hemisphere) to appreciate the full extent of the stimulated cortical area at which peak relaxation rate remains similar as compared to stimulation at the vertex. Relaxation was measured twice for each position in random order, with the TMS coil centred over the position on the cap. During this part of the protocol, exerted force was 50% MVC, in order to avoid fatigue due to the many contractions.

*Temperature (day 3) –* TMS‐induced muscle relaxation was first measured three times at start temperature (i.e., before cooling or warming). Afterwards, the subject's forearm was submerged for 15 min in cold water (10°C). This was done by placing the forearm horizontally (elbow in 90° angle) in a water bath with the water level at 5 cm proximal of the elbow joint. Relaxation measurements were acquired every 30s during the following 10 min, then every minute for another 20 min, while the arm gradually warmed up. This was followed by submerging the subject's forearm in warm water (40°C) for 10 min, and measurements were repeated every 30s during the following six minutes. Target force for this experiment was 50% MVC to avoid fatigue induced slowing of relaxation properties.

### 2.5 Data analysis

**FIGURE 3:**
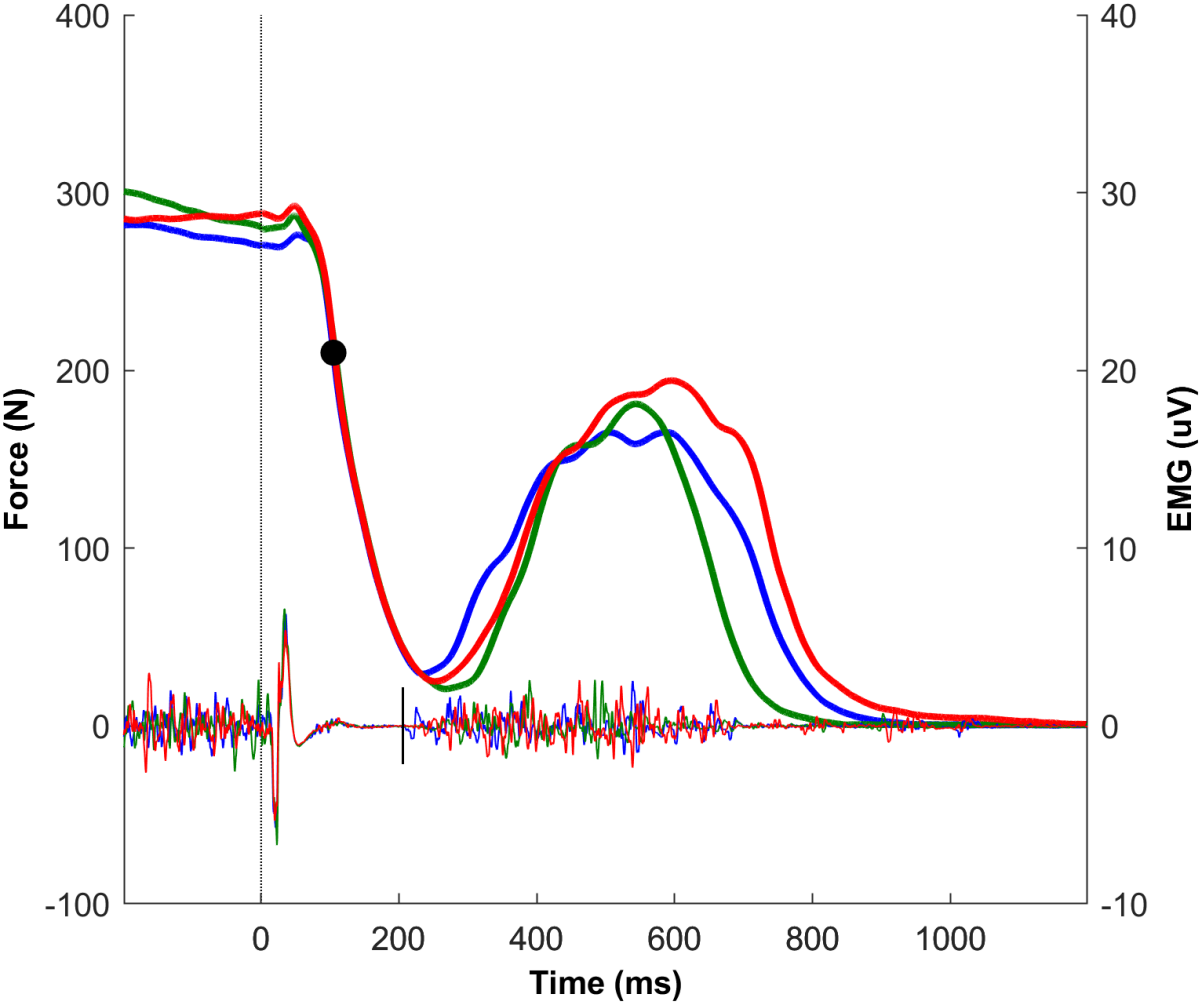
Force and EMG curves of TMS‐induced muscle relaxation. Example of three repeat force (above, left *y*‐axis) and EMG (below, right *y*‐axis) curves from one subject. In the force curve, peak relaxation rate (black filled circle) is visualized. The dotted line represents the moment of TMS‐pulse (*t* = 0). The end of the silent period in the EMG signal is marked by the solid line. Note the limited variation in the relaxation phase of the force curves

The peak relaxation rate of the deep finger flexors was defined as the steepest negative slope in the force curve during the TMS‐induced silent period (Figure 3). This peak rate of force decline was then normalized to the peak force (voluntary plus TMS evoked) preceding the silent period (de Ruiter, Jones, et al., 1999; Ranatunga, 1982). Silent period duration was defined as the time from the TMS pulse to the return of voluntary EMG (visually assessed) (McNeil et al., 2013; Taylor et al., 1997).

### 2.6 Statistical analysis

Normality of the data was tested with the Shapiro–Wilk normality test. Interday and interrater reproducibility was assessed by calculating the coefficient of variation (CV), defined as the ratio of the standard deviation (SD) to the mean. To quantify interday and interrater reliability, the intraclass correlation coefficient (ICC) was calculated (Bartlett & Frost, 2008). For interday reliability, we used a two‐way mixed model, type absolute agreement. For interrater reliability, we used a two‐way random model, type absolute agreement (Koo & Li, 2016). For the intra(within)‐rater ICC, a one‐way random model was used for each rater separately. We tested for a fatigue effect on day 1, that is, the most strenuous day (20 MVCs on day 1 vs 9 and 3 MVCs on day 2 and 3, respectively) by comparing the average of the first 3 MVCs to the last 3 MVCs for all subjects with a two‐sided paired *t*‐test.

To assess robustness, repeated measures ANOVA and Tukey's post hoc test was performed to identify the effects of exerted force (%MVC), stimulus intensity (%MSO) and TMS coil position. To compare one condition to the default condition (i.e., stimulus over vertex during a 100% MVC with %MSO that induced a silent period of ~200 ms) a post‐hoc Dunnett's multiple comparisons test was performed. ANOVA results are displayed as F‐value with the degrees of freedom set as subscript, followed by the *p*‐value.

For the temperature curves a Bolzmann sigmoid curve was fitted through all datapoints for all individual subjects (Prism, GraphPad Software, version 5.03). To calculate the effect of skin temperature on relaxation rate within the range of normal skin temperatures, the average increase in normalized peak relaxation rate of the fitted sigmoid curve between the lowest and highest baseline skin temperature was calculated for all individual subjects (Figure 7). Additionally, we tested the effect of muscle cooling and heating on normalized peak relaxation rate by comparing the three baseline measurements with the first three measurements after cooling and the first 3 measurements after heating using repeated measures ANOVA with Tukey's multiple comparisons test. Temperature effects in our interday variation study were quantified using Pearson correlation.

Data are described as mean ± standard deviation, unless mentioned otherwise. *p*‐values <0.05 were considered statistically significant, all probability values were two‐sided. Statistical analyses were performed using Graphpad Prism and SPSS (IBM SPSS Statistics, version 22). Anonymized data and Matlab algorithms are available on request via the corresponding author.

## 3 RESULTS

Baseline characteristics of the subjects can be found in Table 2. All participants were categorized as *active* (score = 4) according to the activity questionnaire.

**TABLE 2:** Subject characteristics

	Mean ± SD (range)
Sex (M/F)	5/5
Age (years)	25.7 ± 3.5 (21.7–33.2)
Height (cm)	176.8 ± 6.0 (166.0–186.0)
Weight (kg)	73.9 ± 11.0 (61.7–94.7)
BMI (kg/m^2^)	23.6 ± 2.9 (20.6–28.6)
MVC (N)*	470 ± 126 (310–664)

*Note*: Values are mean ± standard deviation.

Abbreviations: BMI, body mass index; MVC, Maximal voluntary contraction; N, Newton.

* Strongest contraction on day 1 prior to any TMS‐stimulation.

### 3.1 Interday variation

The interday period was 7.3 ± 1.5 days (range 5–11 days). Differences in normalized peak relaxation rate between days (day 1 – day 2) were normally distributed and not different from zero (average 0.2 ± 0.1 s^−1^, *p* = 0.50), which indicates no bias between days. The same holds for the averaged silent period (*p* = 0.36) and MVC (*p* = 0.72). Coefficients of variation (CV) and intraclass correlation coefficients (ICC) for all parameters are listed in Table 3. Differences in skin temperature between days were weakly correlated with differences in normalized peak relaxation rate (*r* = 0.477, *p* = 0.04).

**TABLE 3:** Interday and interrater reproducibility and reliability

	Day 1 (*n* = 10)	Day 2 (*n* = 10)	CV (%)	ICC	Rater A (*n* = 10)	Rater B (*n* = 10)	CV (%)	ICC
NpRR (s^−1^)	13.1 ± 2.0	12.8 ± 1.9	3.8 ± 4.0	0.88 [0.59–0.97]	13.0 ± 1.9	12.7 ± 1.9	3.7 ± 2.3	0.92 [0.72–0.98]
MVC (N)	444.6 ± 133.4	437.7 ± 96.7	6.6 ± 5.2	0.88 [0.60–0.97]	431.9 ± 98.6	438.0 ± 100.9	2.9 ± 2.8	0.98 [0.93–1.00]
Silent period (ms)	218.3 ± 18.8	212.3 ± 15.4	4.1 ± 4.8	0.35 [−0.31–0.78]	212.1 ± 18.2	216.0 ± 14.5	3.7 ± 2.9	0.63 [0.06–0.89]
Stimulus intensity (%MSO)	77.0 ± 10.6	75.0 ± 10.8	1.9 ± 4.0	0.91 [0.70–0.98]	75.0 ± 10.8	73.0 ± 10.6	1.9 ± 4.0	0.91 [0.70–0.98]

*Note*: Values are mean ± standard deviation or estimate [95% confidence interval].

Abbreviations: CV, coefficient of variation; ICC, intraclass correlation coefficient; MSO, maximal stimulator output; MVC, maximum voluntary contraction; N, Newton; NpRR, normalized peak relaxation rate.

### 3.2 Inter‐ and intrarater variation

Interrater CV and ICC (reproducibility and reliability between raters) for all parameters are listed in Table 3. Differences in normalized peak relaxation rate between rater A and rater B were normally distributed and not different from zero (average − 0.26 ± 0.77, *p* = 0.31), which indicates no significant bias between the two raters. The same applies for the averaged silent period (*p* = 0.41) and MVC (*p* = 0.45). The applied stimulus intensity (to induce a silent period of approximately 200 ms) was the same for both raters in eight out of ten subjects. In the other two subjects, intensity differed by 10% (one step). The last MVC with the first rater and the first MVC with the second rater were separated by 23.6 ± 25.1 min (range 10.5–86.5 min).

The repeated measures (three times TMS‐induced relaxation per subject) of the raters were analyzed to determine the intrarater (i.e., within‐subject) repeatability and reliability. For normalized peak relaxation rate, the intrarater CVs were 3.6 ± 2.5% (Rater A) and 3.3 ± 1.8% (Rater B) and the intrarater ICC was 0.98 for both raters (95% CI for Rater A: 0.93–0.99, Rater B: 0.94–0.99), indicating good to excellent repeatability and reliability for both individual raters.

On day 1 there was no difference between the first 3 MVCs and the last 3 MVCs of the protocol (444.6 ± 133.4 N vs. 416.3 ± 103.5 N, respectively; *p* = 0.08), which implies no significant fatiguing effect of the repeated MVCs.

### 3.3 Target contractile force

**FIGURE 4:**
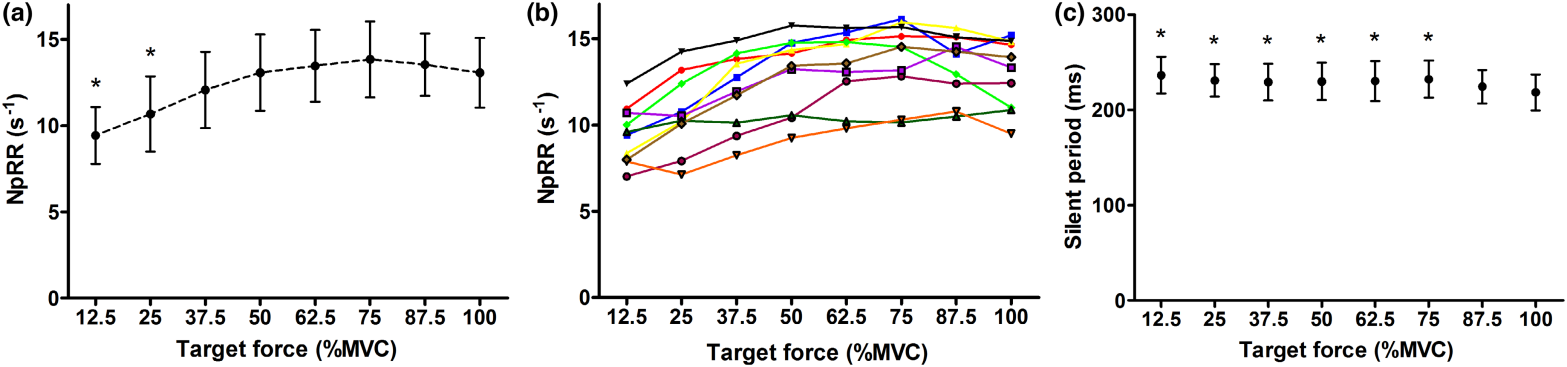
Effect of target contractile force on normalized peak relaxation rate, *n* = 10. Grouped (a) and individual (b) data are presented. There is no further increase in normalized peak relaxation rate with target contractile force ≥37.5% MVC. Panel C demonstrates the effect of target contractile force on the silent period duration. Error bars depict SD. MVC, Maximal voluntary contraction; NpRR, normalized peak relaxation rate. *Significantly different from 100%MVC (*p* < 0.05)

There was a significant influence of target force (%MVC) on normalized peak relaxation rate (F_7,9_ = 24.0, *p* < 0.0001) and on silent period duration (F_7,9_ = 6.4, *p* < 0.0001). Only in the lowest two conditions (12.5% and 25% MVC) normalized peak relaxation rate differed significantly from 100% MVC (Figure 4). Silent period duration was longer in target force levels of 12.5–75% MVC compared to 100% MVC (Figure 4c). There was a significant effect of target force on the start of the muscle relaxation phase (F_7,9_ = 46.8, *p* < 0.0001) with the lower force targets having a longer time between the TMS pulse and top of the superimposed twitch (84.5 ± 5.8 ms vs 60.7 ± 10.4 ms for 12.5% MVC and 100% MVC, respectively; *p* < 0.0001).

### 3.4 Stimulator output intensity

**FIGURE 5:**
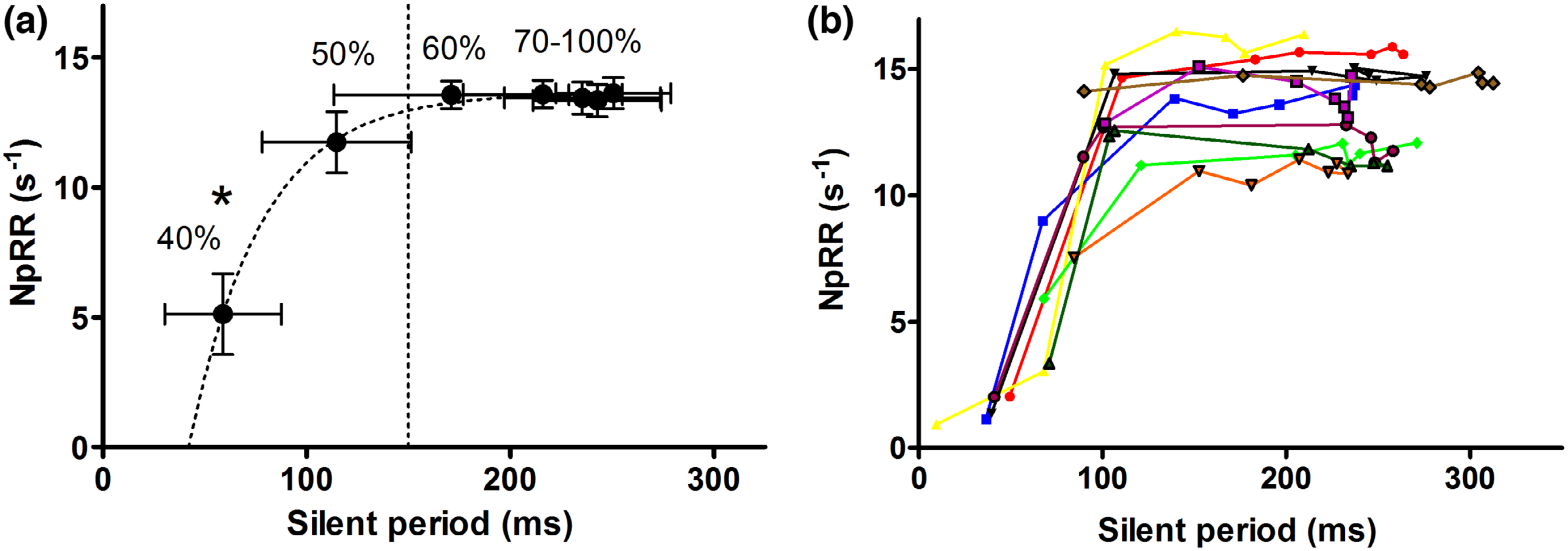
Effect of silent period duration on normalized peak relaxation rate, *n* = 10. Grouped (a) and individual (b) data are presented. In panel A, the silent period duration is visualized for all used TMS intensities (%MSO depicted next to the mark). Note the stabilization and decreasing SD of the normalized peak relaxation rate with stimulus intensity ≥60%MSO and silent period >150 ms (dotted vertical line). Error bars depict SD. *Significantly different from normalized peak relaxation rate at 100%MSO (*p* < 0.05). MSO, maximal stimulator output; NpRR, normalized peak relaxation rate

There was a significant effect of stimulator output intensity on normalized peak relaxation rate (F_6,9_ = 15.5, *p* < 0.0001). Only a stimulator output of 40% MSO resulted in significantly lower normalized peak relaxation rate compared to all other intensities (*p* < 0.0001) (Figure 5a). Normalized peak relaxation rates stabilized after a silent period duration of >150 ms (Figure 5b, vertical dotted line). This is consistent with an average time to peak relaxation rate of 107.4 ± 6.5 ms, range 99.8–117.3 ms (time between TMS pulse and the timepoint of peak relaxation rate).

### 3.5 TMS coil position

**FIGURE 6:**
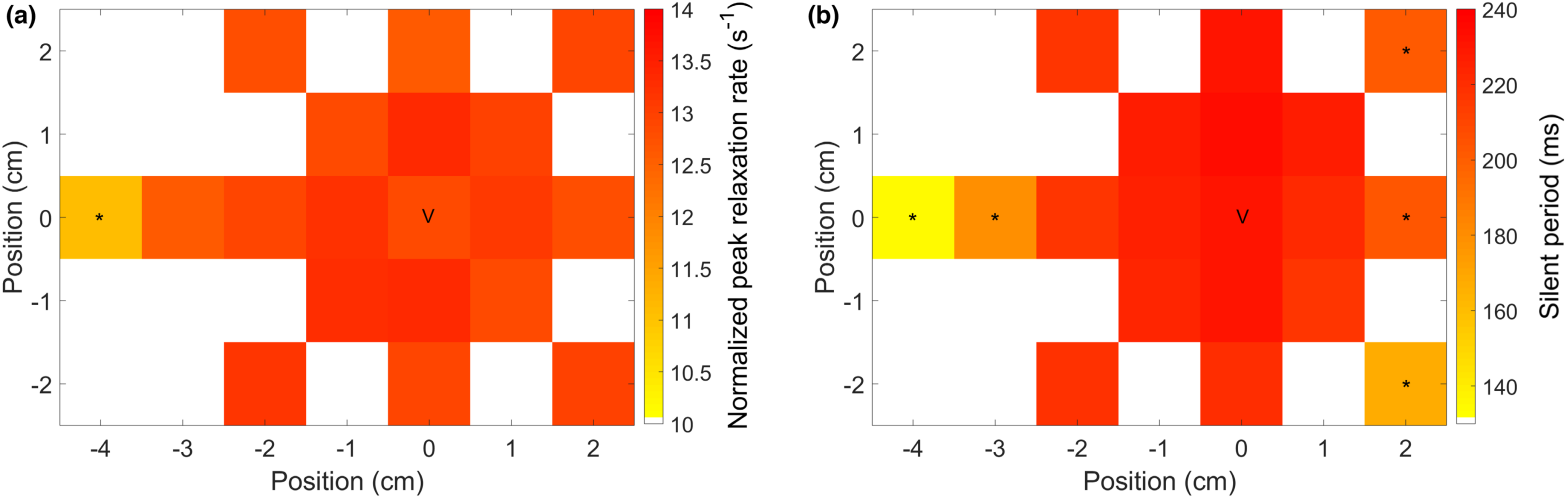
Heatmap of the effect of TMS coil position on normalized peak relaxation rate (a) and silent period duration (b), *n* = 10. Only the most lateral position had a significantly lower normalized peak relaxation rate and a silent period <150 ms. *Significantly different from measurement at the vertex (v) (*p* < 0.05)

There was a significant effect of TMS coil position on normalized peak relaxation rate (F_18,9_ = 3.7, *p* < 0.0001) and silent period duration (F_18,9_ = 20.1, *p* < 0.0001). Only stimulation at the most lateral position rendered a significantly lower normalized peak relaxation rate compared to stimulation at the vertex (*p* < 0.0001, see Figure 6a). Regarding the silent period, the most lateral positions on both sides were different with respect to the vertex stimulation (see Figure 6b).

### 3.6 Temperature

**FIGURE 7:**
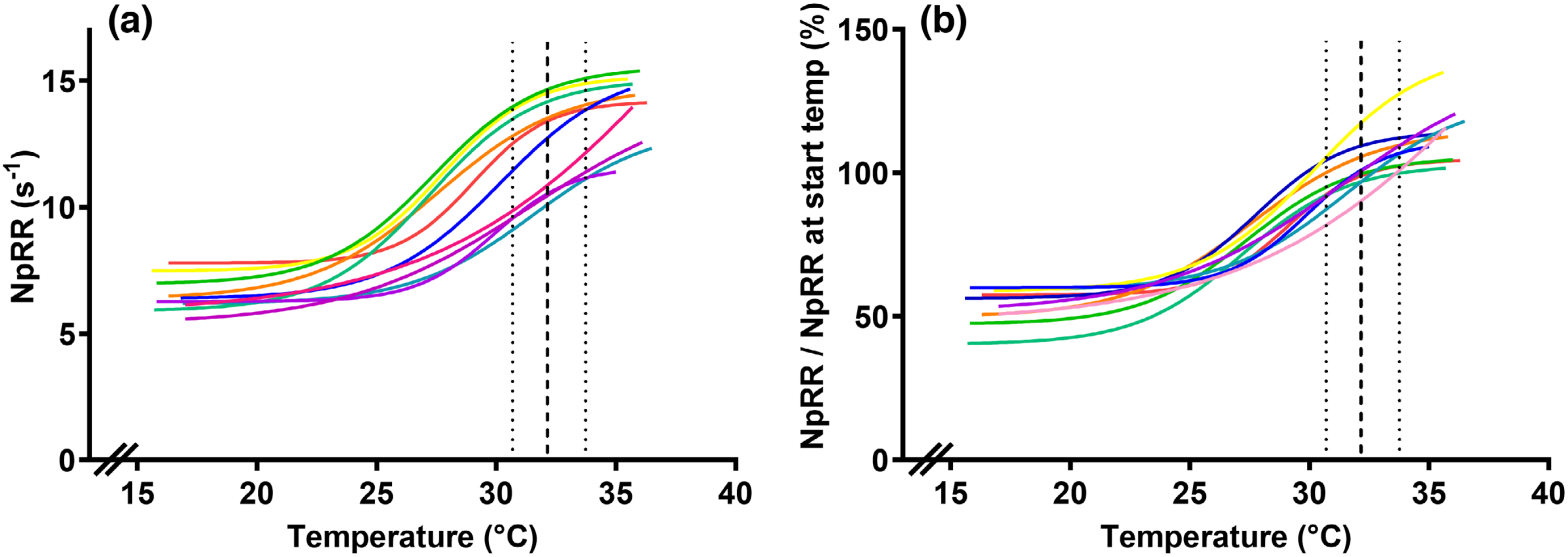
Effect of skin temperature on normalized peak relaxation rate, *n* = 10. Each line represents an individual subject's fitted Boltzmann sigmoid curve. Panel A shows muscle relaxation as a percentage of normalized peak relaxation rate at baseline temperature, panel B shows absolute normalized peak relaxation rate. Figure S1 [https://doi.org/10.6084/m9.figshare.19181900] shows all data points that generated these curves. Goodness of fit of the Boltzmann curves was very good for all individual subjects (*R* ^2^ ranging from 0.94 to 0.99). The dashed line depicts the average initial skin temperature. The dotted lines depict the range of baseline skin temperatures. The average increase in normalized peak relaxation rate of the fitted sigmoid curves between these limits (30.6–33.7°C) was calculated for all individual subjects to assess the average effect of skin temperature on relaxation rate within the range of normal skin temperatures. NpRR at start temp = average normalized peak relaxation rate of the three measurements at baseline temperature (before cooling/heating)

Baseline skin temperature (before cooling) was 32.1 ± 0.9°C (range 30.6–33.7°C). All individual Bolzmann curves of normalized peak relaxation rate against skin temperature are displayed in Figure 7. A sigmoid curve was chosen because this had the best fit of all linear/non‐linear fits. Figure S1 [https://doi.org/10.6084/m9.figshare.19181900] shows all data points that generated these curves. Within the range of baseline (normal) skin temperatures, an average change of 0.5 ± 0.2 s^−1^ (=4.4 ± 1.9% change from baseline) in normalized peak relaxation rate was seen per 1 °C change in skin temperature.

Additionally, we compared muscle relaxation at baseline temperature to the first three measurements after cooling and the first three measurements after heating, that is, at the lowest and highest skin temperatures, respectively. This showed that normalized peak relaxation was slower after cooling (6.6 ± 0.8 s^−1^; *p* ≤ 0.0001) and faster after heating (13.6 ± 1.3 s^−1^; *p* = 0.010) compared to relaxation rate at baseline skin temperature (vs. 12.3 ± 1.7 s^−1^).

There was a limited effect of skin temperature on silent period duration with an average increase in silent period duration of 0.38 ± 0.17 ms per 1°C change in skin temperature (*p* = 0.02, *R* ^2^ = 0.01).

## 4 DISCUSSION

In this study, we demonstrated that TMS‐induced muscle relaxation has a high interday and interrater reproducibility and reliability. Furthermore, muscle relaxation rate is robust over a large range of target contractile force, stimulator output, and coil position. Low skin temperature has a large effect on muscle relaxation rate, but within the normal range of skin temperature relaxation rate remains similar. Consequently, TMS‐induced muscle relaxation is a very reproducible and robust technique to study muscle relaxation kinetics.

### 4.1 Interday and interrater reproducibility and reliability

Interday and interrater reproducibility and reliability of TMS‐induced muscle relaxation were (very) high, indicated by low CVs and high ICCs, respectively (Fujimura et al., 2013; Koo & Li, 2016). Furthermore, we found no evidence of bias between days or raters. Part of the interday variation in normalized peak relaxation rate can be explained by a difference in skin temperature between days. The intrarater (within‐subject) repeatability and reliability are in accordance with our previous study on TMS‐induced muscle relaxation in finger flexor muscles (Molenaar et al., 2018). Recently, repeatability of TMS‐induced muscle relaxation was also studied in knee‐extensor muscles which also demonstrated high repeatability and reliability (Vernillo et al., 2021). The demonstrated high repeatability (small within‐subject variability) and reliability in these previous studies and our current study lead to high statistical power when studying the effect of an intervention on muscle relaxation or to detect differences in relaxation rate between groups for a given sample size (Bartlett & Frost, 2008). Therefore, this technique is particularly well suited to study muscle relaxation in rare diseases such as myopathies (Molenaar et al., 2018).

### 4.2 Target contractile force

Low contraction force (12.5% and 25% MVC) resulted in a lower normalized peak relaxation rate as compared to 100% MVC even though silent period duration was longer at lower target force levels. This could be explained by the higher proportion of slow‐twitch muscle fibers that are recruited at lower levels of activation (Henneman, 1957; Jabre & Spellman, 1996). When studying maximal normalized peak relaxation rates we recommend instructing the subject to use maximum force and not to worry if force varies between measurements (only a force of <37,5% MVC will significantly decrease normalized peak relaxation rate). When a larger number of measurements are gathered we recommend using a target force of 50% MVC to eliminate fatigue induced slowing of normalized peak relaxation rate.

### 4.3 Stimulator output intensity

We found no difference in normalized peak relaxation rate with a stimulator output of 50% MSO and higher (Figure 5a). This can be explained by the relation between the stimulator output and the duration of the induced silent period. With increasing stimulator output there was an increased corticospinal inhibition (i.e. increased silent period, Figure 5b), as demonstrated previously (Kojima et al., 2013; Saisanen et al., 2008). A stimulus intensity that induced a silent period of 150 ms or more was sufficient to elicit maximal normalized peak relaxation rate (Figure 5b). A longer inhibition phase, induced by a higher stimulus intensity, did not result in faster relaxation (only longer relaxation). Silent periods of less than 150 ms should be regarded as unreliable for maximum relaxation measurements and should therefore be excluded. These findings concur with a previous study on the effects of TMS intensity on relaxation rates in lower leg muscles (McNeil et al., 2013).

### 4.4 Coil position

Within a square of 2x2 cm surrounding the vertex, stimulation with sufficient intensity resulted in similar normalized peak relaxation rate (Figure 6). Circular TMS coils have a strong, non‐focal field that induce electrical currents in a large volume of brain tissue (Groppa et al., 2012). This allows for minor displacements in coil position, without compromising the quality of the relaxation measurements. Therefore, very precise stimulation at the vertex is not necessary to sufficiently inhibit the cortical representation of finger flexor muscles, which saves preparation time in a clinical setting. More precise targeting of the large hand/forearm cortical area (using a more focal coil type) could even underestimate relaxation rate as maximal relaxation speed requires inhibition of all involved motor units. Only the most lateral position had a significantly lower normalized peak relaxation rate and a silent period <150 ms (Figure 6). This result matches our finding that a silent period below 150 ms results in submaximal peak relaxation rate. Generalizing these results to other muscle groups should be done with some caution as other cortical motor areas (e.g., leg muscles) might need more precise targeting using a double‐cone coil (McNeil et al., 2013).

### 4.5 Temperature

Previous studies have shown that temperature has a profound effect on muscle relaxation properties, e.g. (de Ruiter, Jones, et al., 1999; Hopf & Maurer, 1990; Molenaar et al., 2018; Todd et al., 2007). To our knowledge, this is the first in vivo study to assess this effect in such detail and demonstrate a non‐linear (sigmoid) relationship between skin temperature and relaxation rate. Muscle relaxation rate was greatly influenced by cooling with a 50% decrease in normalized peak relaxation rate at the lower temperature range (skin temperature ~ 16°C). This is similar to what other studies showed in different muscles (De Ruiter & De Haan, 2000; de Ruiter, Jones, et al., 1999; Hopf & Maurer, 1990).

Normalized peak relaxation rate increased by ~10% after muscle heating compared to baseline skin temperature. This is in line with previous research demonstrating faster TMS‐induced muscle relaxation in heated muscle with the use of intramuscular temperature assessment (Todd et al., 2005; Todd et al., 2007).

Within the range of baseline skin temperatures, the temperature effect on muscle relaxation was limited. Similar to nerve conduction velocity studies, we recommend warming the forearm in a warm water bath of 36°C for 30 min when initial skin temperature (measured at forearm) is below 32°C (Franssen & Wieneke, 1994). When this technique is used to monitor a subject over time, we recommend avoiding large temperature differences. In our study skin temperatures were not controlled to study interday reproducibility. Differences in skin temperature between days correlated with differences in normalized peak relaxation rate, thus explaining part of the interday variation.

We used skin temperature as a substitute for muscle temperature as it is more practical and patient friendly as compared to invasive intramuscular temperature measurement. Additional research is required to test if our results hold true for intramuscular temperature and peak relaxation rate.

### 4.6 Clinical significance

Impaired muscle relaxation is a main feature in several neuromuscular disorders, such as myotonic dystrophy or Brody disease (Matthews et al., 2010; Odermatt et al., 1996). We demonstrated that TMS‐induced muscle relaxation results in reliable and repeatable measurements, and is robust with respect to changes in several experimental conditions. This makes the technique very suitable to apply in a clinical setting and for multicenter studies on relaxation kinetics in healthy and affected muscle. It could be a valuable asset in the diagnosis of neuromuscular diseases, disease progression monitoring, and as an outcome measure in clinical trials. It should be noted that our subjects were healthy young adults. Therefore, extrapolating our results and recommendations to other populations such as myopathy subjects or the elderly should be done with some caution.

### 4.7 Conclusions and recommendations

TMS‐induced muscle relaxation of finger flexors is a highly reproducible and robust technique to assess muscle relaxation kinetics in clinical practice and experimental studies. We recommend to apply this technique with a stimulation strength that induces a silent period of minimally 150 ms; a round coil within 2x2 cm surrounding the vertex; and target force of ≥50% of MVC. Furthermore, we recommend monitoring skin temperature, warming the forearm in a warm water bath of 36°C for 30 min when initial skin temperature is below 32°C, and minimizing temperature differences between measurements.

## AUTHOR CONTRIBUTIONS

J.P.M., E.v.Z, B.G.v.E., N.C.V., and J.D. conceived and designed research; J.P.M., E.v.Z, and J.D. performed experiments; J.P.M., E.v.Z, and J.D. analyzed data; J.P.M., E.v.Z, B.G.v.E., N.C.V., and J.D. interpreted results of experiments; J.P.M., E.v.Z, and J.D. prepared figures; J.P.M. and E.v.Z drafted manuscript; B.G.v.E., N.C.V., and J.D. edited and revised manuscript; J.P.M., E.v.Z, B.G.v.E., N.C.V., and J.D. approved final version of manuscript.

## FUNDING INFORMATION

None.

## CONFLICT OF INTEREST

None.

## Supporting information


Figure S1
