# Supplementary figures and images for: Reproducibility and robustness of motor cortical stimulation to assess muscle relaxation kinetics
Source: Physiol Rep. 2022 Oct 20;10(20):e15491. doi: 10.14814/phy2.15491 (PMC9585355; doi:10.14814/phy2.15491)

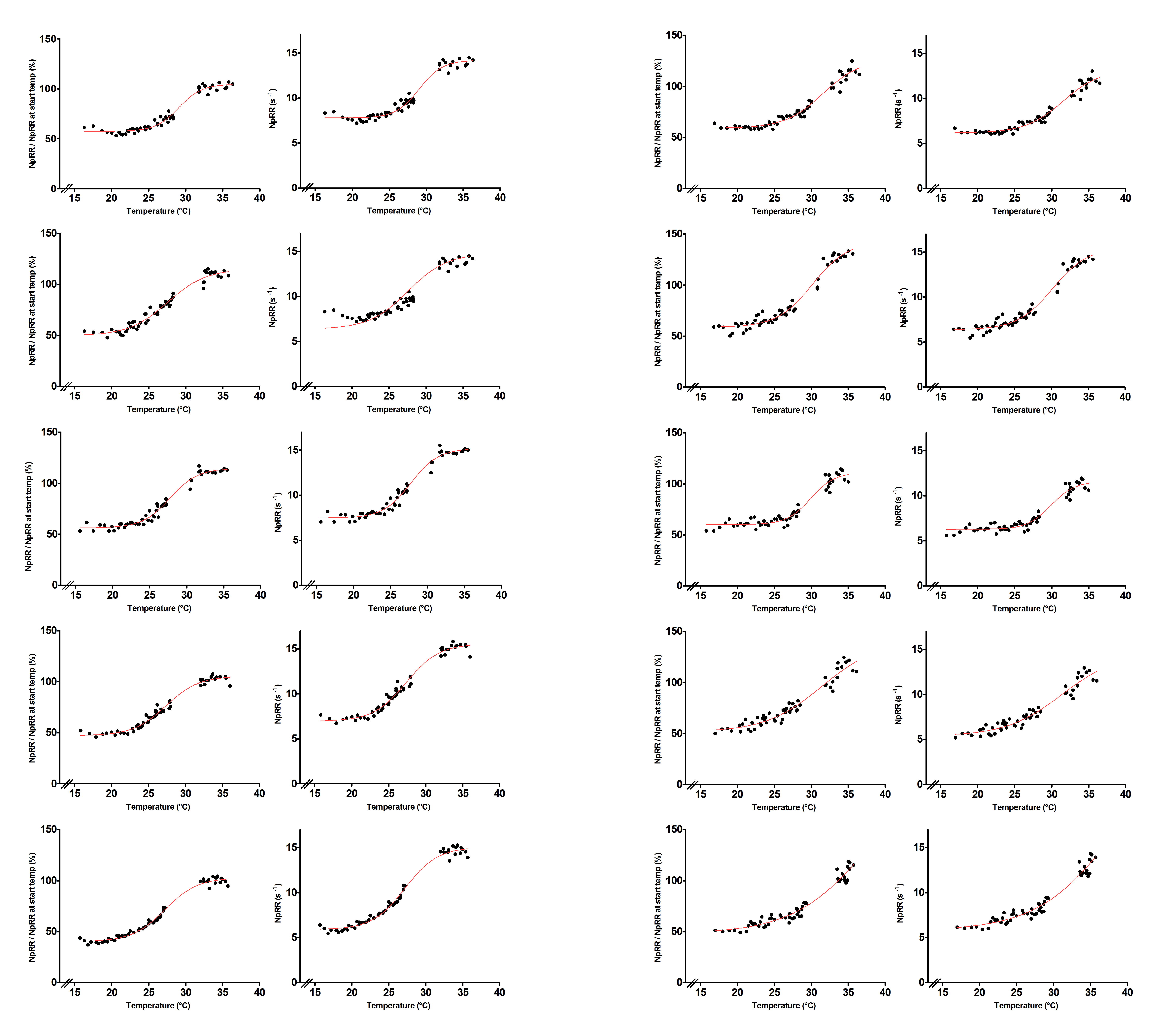

Supplement: Supplementary file 1 — Figure S1 [file PHY2-10-e15491-s001.tif]
